# 2207. Rates of Lower Respiratory Tract Infections Among US Adults Aged ≥18 Years With and Without Chronic Medical Conditions

**DOI:** 10.1093/ofid/ofac492.1826

**Published:** 2022-12-15

**Authors:** Derek Weycker, Ahuva Averin, Linnea Houde, Kevin Ottino, Kimberly M Shea, Bradford D Gessner, Kari Yacisin, Daniel Curcio, Elizabeth Begier, Mark Rozenbaum

**Affiliations:** Policy Analysis Inc., Chestnut Hill, Massachusetts; Policy Analysis Inc., Chestnut Hill, Massachusetts; Policy Analysis Inc., Chestnut Hill, Massachusetts; Policy Analysis Inc., Chestnut Hill, Massachusetts; Pfizer Inc., Newton, Massachusetts; Pfizer Biopharma Group, Collegeville, Pennsylvania; Pfizer Inc., Newton, Massachusetts; Pfizer Inc., Newton, Massachusetts; Pfizer Vaccines, Dublin, Dublin, Ireland; Pfizer Inc., Newton, Massachusetts

## Abstract

**Background:**

While it is widely recognized that older adults and adults with chronic medical conditions are at increased risk of lower respiratory tract infections (LRTI), available evidence on the magnitude of increased risk is limited.

**Methods:**

A retrospective observational cohort study using IBM MarketScan Commercial/Medicare Databases (2016–2019) was conducted. The study population included all adults (age ≥ 18 years) and was stratified by age and comorbidity profile (with vs. without high-risk conditions, based on recommendations for influenza vaccination in the United States). LRTI was ascertained on an overall basis as well as by causative pathogen (e.g., respiratory syncytial virus [RSV]) based on corresponding diagnosis codes, and was classified based on care setting (hospital, emergency department [ED], physician office/hospital outpatient [PO/HO]). Incidence rates (and relative rates [RRs]) were generated by age, and within each age group, by comorbidity profile.

**Results:**

Using adults aged 18-34 years as the reference, RR of LRTI generally increased with older age across care settings, with the most marked increase for hospitalizations: for hospitalized-LRTI, RRs ranged from 1.7 for 35-49 years to 78.9 for ≥ 85 years; for ED-LRTI and PO/HO-LRTI, RRs ranged from 1.0 to 3.4 and from 1.4 to 2.1, respectively (Table). Within age groups, LRTI rates were also consistently higher among adults with versus without high-risk conditions: for hospitalized-LRTI, RRs ranged from 9.9 to 21.1; for ED-LRTI, from 2.3 to 3.2; and for PO/HO-LRTI, from 1.6 to 2.5. Age-specific RRs of hospitalized-LRTI due to RSV were largely comparable to overall LRTI results; age-specific RRs for other care settings, and RRs for adults with versus without high-risk conditions across care settings, were generally higher for LRTI due to RSV.

**Figure:**
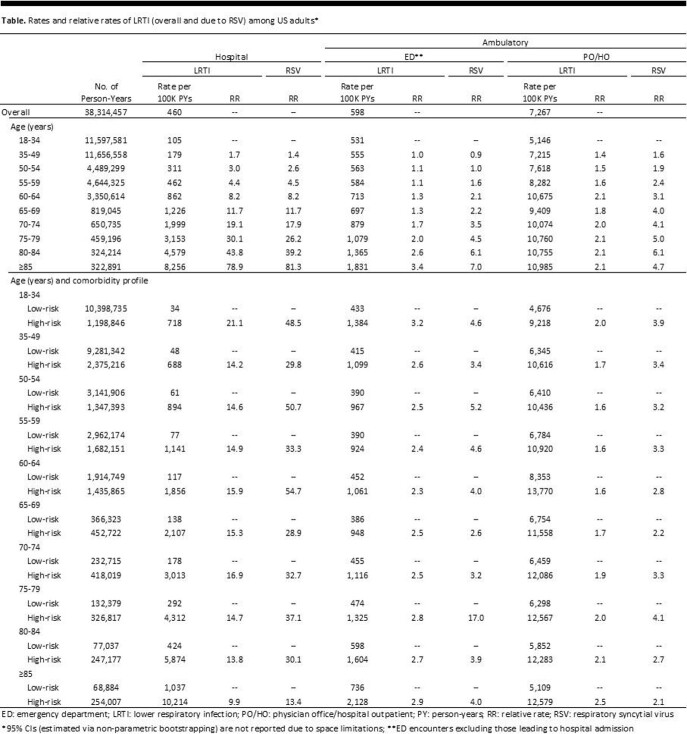

**Conclusion:**

LRTI incidence, especially for events requiring acute inpatient care, is markedly higher among older adults and adults of all ages with chronic medical conditions. Effective vaccines against respiratory pathogens could help reduce this elevated risk of LRTI.

**Disclosures:**

**Derek Weycker, Ph.D.**, Pfizer Inc.: Grant/Research Support **Ahuva Averin, M.P.P.**, Pfizer Inc.: Grant/Research Support **Linnea Houde, M.S.**, Pfizer Inc.: Grant/Research Support **Kevin Ottino, M.H.S.**, Pfizer Inc.: Grant/Research Support **Kimberly M. Shea, Ph.D., M.P.H.**, Pfizer: Employee|Pfizer: Stocks/Bonds **Bradford D. Gessner, M.D., M.P.H.**, Pfizer Inc.: Employee|Pfizer Inc.: Stocks/Bonds **Kari Yacisin, M.D.**, Pfizer Inc.: Employee|Pfizer Inc.: Stocks/Bonds **Daniel Curcio, M.Sc.**, Pfizer Inc.: Employee|Pfizer Inc.: Stocks/Bonds **Elizabeth Begier, M.D., M.P.H.**, Pfizer: Employee|Pfizer: Stocks/Bonds **Mark Rozenbaum, Ph.D., M.B.A.**, Pfizer Inc.: Employee|Pfizer Inc.: Stocks/Bonds.

